# Defining the landscape of metabolic dysregulations in cancer metastasis

**DOI:** 10.1007/s10585-021-10140-9

**Published:** 2021-12-18

**Authors:** Sara Abdul Kader, Shaima Dib, Iman W. Achkar, Gaurav Thareja, Karsten Suhre, Arash Rafii, Anna Halama

**Affiliations:** 1 Department of Physiology and Biophysics, Weill Cornell Medicine-Qatar, 24144 Doha, Qatar; 2 University of Paris-Saclay, 91190 Gif-sur-Yvette, France; 3 Department of Biophysics and Physiology, Weill Cornell Medicine, New York, USA; 4 Department of Genetic Medicine, Weill Cornell Medicine, New York, USA; 5 Genetic Intelligence Laboratory, Weill Cornell Medicine in Qatar, Qatar Foundation, Doha, Qatar

**Keywords:** Metastasis, Metastatic potential, Triple negative breast cancer, Metabolic profiling, TCA cycle, Branch chain amino acid metabolism

## Abstract

Metastasis is the primary cause of cancer related deaths due to the limited number of efficient druggable targets. Signatures of dysregulated cancer metabolism could serve as a roadmap for the determination of new treatment strategies. However, the metabolic signatures of metastatic cells remain vastly elusive. Our aim was to determine metabolic dysregulations associated with high metastatic potential in breast cancer cell lines. We have selected 5 triple negative breast cancer (TNBC) cell lines including three with high metastatic potential (HMP) (MDA-MB-231, MDA-MB-436, MDA-MB-468) and two with low metastatic potential (LMP) (BT549, HCC1143). The normal epithelial breast cell line (hTERT-HME1) was also investigated. The untargeted metabolic profiling of cells and growth media was conducted and total of 479 metabolites were quantified. First we characterized metabolic features differentiating TNBC cell lines from normal cells as well as identified cell line specific metabolic fingerprints. Next, we determined 92 metabolites in cells and 22 in growth medium that display significant differences between LMP and HMP. The HMP cell lines had elevated level of molecules involved in glycolysis, TCA cycle and lipid metabolism. We identified metabolic advantages of cell lines with HMP beyond enhanced glycolysis by pinpointing the role of branched chain amino acids (BCAA) catabolism as well as molecules supporting coagulation and platelet activation as important contributors to the metastatic cascade. The landscape of metabolic dysregulations, characterized in our study, could serve as a roadmap for the identification of treatment strategies targeting cancer cells with enhanced metastatic potential.

**Supplementary Information:**

The online version contains supplementary material available at 10.1007/s10585-021-10140-9.

## Introduction

Metastatic disease accounts for approximately 90% of cancer related deaths [1, 2], despite relatively low metastatic efficiency due to the challenging multistep cascade required to establish colonies in distant tissue [3]. Triple negative breast cancer (TNBC), characterized by the lack of expression of the estrogen receptor (ER), progesterone receptor (PR) and human epidermal growth factor receptor 2 (HER2) [4], tends to display a more aggressive clinical course with frequent distant recurrence and thus poor prognosis compared to other breast cancer types [5]. Lack of available targeted therapy for TNBC patients along with the limited understanding of the molecular processes governing metastatic disease reflect on very narrow treatment options for those patients [6]. Therefore, further insights into molecular events related to metastasis could revel novel treatment targets.

Metabolomics, provides almost unbiased overview of the current processes that are ongoing in the biological system by monitoring the levels of endogenous and exogenous small molecules (metabolites) in that system [7]. Hence, metabolic profiling can precisely inform on altered molecular pathways and responses to environmental stimuli. The metabolic signatures discriminating healthy from disease are frequently deployed for biomarkers identification but also to provide insights into the pathological processes causing disease [8–10]

In the last decade, our view on cancer as being a strictly genetic disease has evolved and nowadays, cancer is also considered as a metabolic disorder [11]. This insight arises from the vast body of evidence from multiple studies showing drastic differences between metabolism of cancer and normal cells in glycolysis, glutaminolysis, nucleotide metabolism, as well as synthesis and catabolism of lipids [12–16]. The metabolic dysregulations observed in cancer cells can became a basis for new drug discoveries [10], designed to target cancers with e.g. enhanced glutaminolysis [17] and fatty acid synthesis [18], as well as for the identification of cancer survival mechanisms under treatment [19–21]

Recently, cancer metabolic plasticity related to the ability of cancer cells to fulfill the metabolic requirements of the metastatic cascade, as well as metabolic flexibility related to cancer cells’ use of different nutrients to meet the energetic requirements during metastasis, were defined as key contributors enabling cancer cell adjustment during metastasis [22]. The impact of metabolic rewiring on metastatic signaling cascade was also suggested [23]. For instance, dysregulations in tricarboxylic acid (TCA) cycle metabolism towards accumulation of fumarate [24] and succinate [25], as well as the generation and accumulation of 2-hydroxyglutarate [26, 27] were linked to DNA methylation and associated with epithelial-mesenchymal transition (EMT) [28]. The role of lipid metabolism as well as glycosylation were defined by us and others as important steps in nesting of cancer cells in the endothelial niche [29, 30]*.* Alterations in metabolism of acetyl-CoA, recognized as an epigenetic regulator for its involvement in histone acetylation, were identified as important components of EMT [31]. However, the metabolic program related to metastatic potential of cancer cell remains largely elusive.

Here, we investigated whether TNBC cell lines harboring different metastatic potential in vivo would differ metabolically in vitro. To this end, we selected five TNBC cell lines (BT549, HCC1143, MDA-MB-231, MDA-MB-436, and MDA-MB-468), for which metastatic potential was recently defined by Jin et al. [32], who created a metastasis map (MetMap), by characterizing organ-specific patterns of metastasis and metastatic potential of 500 different human cancer cell lines from 12 different types of solid tumors, including breast cancer [32]. We used untargeted metabolomics profiling to describe metabolism of TNBC cell lines defined as with low (BT549 and HCC1143) and high (MDA-MB-231, MDA-MB-436, and MDA-MB-468) metastatic potential along with normal epithelial breast cell line (hTERT-HME1). We detected 479 metabolites, which allowed a clear separation between normal and TNBC cell lines on the principal component analysis (PCA) score plot. A total of 291 metabolites displayed significant differences at a false discovery rate (FDR) < 0.01 between normal and TNBC cell lines. Next, we searched for metabolic differences discriminating cell lines with low metastatic potential (LMP) from those with high metastatic potential (HMP), in both, cultured media and cells extracts. We found that cell lines with LMP and HMP are metabolically different, and those differences are independent of canonical EMT markers. We identified enrichment in glycolysis and citrate metabolism as well as enhanced branched chain amino acids (BCAA) catabolism and dysregulated metabolism of lipids as signatures of HMP cell lines. Additionally, we found metabolic features potentially involved in coagulation and platelet activation in HMP cell lines. Our findings shed new light on the landscape of metabolic dysregulations related to the metastatic potential of TNBC cell lines, which could in the further be considered as therapeutic targets.

## Methods

### Culture conditions

The established cancer cell lines (BT-549, HCC1143, MDA-MB-231, MDA-MB-436, MDA-MB-468) all TNBC models and the normal epithelial breast cell line (hTERT-HME1 [ME16C]) were purchased from American Type Culture Collection (ATCC, Manassas, VA, USA). All TNBC cell lines were maintained at 37 °C and 5% CO2 and grown in Roswell Park Memorial Institute medium (RPMI-1640) media supplemented with 10% fetal bovine serum and 1% penicillin–streptomycin. The growth media of BT-549 was supplemented with 1 µg/ml insulin and growth media of MDA-MB-436 with 10 µg/ml insulin and 16 µg/ml glutathione, as per ATCC instructions.

The cell lines dedicated for metabolomics and western blot analysis were cultured and prepared at the same time points on separate Petri dishes with a growth area of 21 cm^2^. The study was conducted in two independent experiments each conducted in triplicates. The cell lines MDA-MB-231, MDA-MB-436, MDA-MB-468 were seeded at the density of 1 × 10^6^ and BT-549 and HCC1143 at the density 1.5 × 10^6^ per dish. 24 h after seeding, the medium was changed with fresh medium and cells were incubated for an additional 24 h. At the day of collection, the cells reached around 85% of confluency. The collection process for metabolomics and for Western blot was conducted 48 h after seeding and the description is provided in Sect. 2.2 and 2.3, respectively.

### Western blot

The medium was aspirated, the cells were washed with phosphate buffered saline (PBS) and incubated for around 1 min with 1 ml of trypsin at 37 °C in the incubator. 1.5 ml media was added to the cells resuspended and placed into a 15 ml tube. The samples were centrifuged for 5 min at 400×*g*, the supernatant was removed, and the cells were resuspended in PBS. The samples were centrifuged, the supernatant was removed, and the cell pellets placed at − 80 °C until further processing.

At the day of processing, the samples were thawed on ice and mixed at the ratio of 1 × 10^6^ cells/50 µl with lysis buffer Nonidet P-40 (NP40) supplemented with protease-phosphatase cocktail inhibitors and phenyl methane sulfonyl fluoride (PMSF). The samples were lysed by three freeze–thaw cycles as previously described [33]. The supernatant was collected after centrifugation for 10 min at 18,000×*g*. The total protein content was quantified using the DC protein assay kit (Bio-Rad, Richmond, CA). The proteins were denatured by incubation with 1 × Laemmli buffer containing β-mercaptoethanol, at 95 °C for 10 min.

The prepared whole cell lysates were used to conduct gel electrophoresis followed by transfer to a polyvinylidene fluoride membrane (Bio-Rad). The membrane was blocked in 5% milk solution in Tween-PBS (PBS with 0.1% Tween 20) for 1 h followed by overnight incubation in a primary antibody at 4 °C. The membrane was washed three times in Tween-PBS and incubated in the corresponding secondary antibody for 1 h at room temperature. The membrane was washed three times prior to development. Both primary and secondary antibodies were prepared in the recommended dilution in a 5% milk or bovine albumin solution in Tween-PBS. The signal was detected by a chemiluminescent western blot detection kit (Thermofischer) and the blots were developed and visualized under a ChemiDoc system (Amersham, Bio-Rad, USA). The primary antibodies used were Twist2 (GeneTex, #GTX50850), MMP-2 (Cell signaling, #40994), Vimentin (Cell signaling, #5741), N-Cadherin (Cell signaling, #13116), E-Cadherin (Cell signaling, #14472), Jagged1 (Cell signaling, #2155), P38 MAPK (Cell signaling, #9212), Akt (Cell signaling, #9272), p-Akt (Cell signaling, #9271), P-p44/42 (Cell signaling, #9106), beta-Tubulin (Cell signaling, #2146s), and beta-Actin (Cell signaling, #3700S). The corresponding secondary antibodies included horseradish peroxidase–conjugated anti-mouse (Cell Signaling) and anti-rabbit (Cell Signaling).

### Sample preparation for metabolic analysis

The growth media was collected into the collection tube, centrifuged for 5 min at 400×*g*, 500 µl was placed into fresh collection tube and flash frozen in liquid nitrogen. The samples were stored at − 80 °C until shipment.

The cell processing for metabolic analysis was conducted as previously described [34]. Briefly, the cells were washed twice with 37 °C PBS. 1 ml of ice-cold 80% methanol in H_2_O was added per dish, and the cells were scraped off from the dish. The scraped-in-methanol cells were placed in a collection tube and flash-frozen in liquid nitrogen, and stored at − 80 °C until further processing. Metabolite extraction out of the cells was conducted in a series of three freeze–thaw cycles; the samples were thawed on ice for 5 min followed by freeze in liquid nitrogen for 5 min. The samples were centrifuged at 18,000×*g* for 5 min at 4 °C, transferred to a fresh collection tube and stored at − 80 °C until shipment.

The remaining pellets were used for the determination of protein content in the samples to account for differences in cell growth. Sample processing was conducted as previously described [35]. Briefly, the remaining pellets were dried in a speed vacuum for 20 min. 60 μl of 0.2 M NaOH was added into the dried samples heated for 20 min at 95 °C with frequent vortexing. The samples were centrifuged at 18,000×*g* for 5 min and the supernatant was transffered to a fresh collection tube. Protein content was determined using the Bio-Rad DC protein assay, relative to bovine serum albumin standards (0–1.8 mg/ml).

The growth media and cell extract were shipped to Metabolon Inc. (Durham, NC, USA) on dry ice for metabolite measurements.

### Metabolic measurements

Metabolic profiling of growth media and cell extracts was performed using Metabolon platforms deploying Waters ACQUITY ultra-performance liquid chromatography (UPLC) and a Thermo Scientific Q-Exactive high-resolution/accurate mass spectrometer interfaced with a heated electrospray ionization (HESI-II) source and Orbitrap mass analyzer, as previously described [36]

Proteins were precipitated from 100 μl of growth media with methanol using an automated liquid handler (Hamilton LabStar). The precipitated extract from growth media and cell extracts were split into four aliquots to undergo the following processes: (1) two fractions for analysis by two separate reverse-phase (RP)/UPLC-mass spectrometry (MS)/MS methods with positive ion mode electrospray ionization (ESI); (2) one fraction for analysis by RP/UPLC-MS/MS with negative ion mode ESI; (3) one fraction for analysis by hydrophilic interaction chromatography (HILIC)/UPLC-MS/MS with negative ion mode ESI. The samples were dried under nitrogen flow.

Dried samples were reconstituted in solvents compatible with each of the four methods: (1) acidic positive ion (optimized for hydrophilic compounds)—extract gradient eluted from a C18 column (Waters UPLC BEH C18–2.1 × 100 mm, 1.7 μm) with water and methanol containing 0.05% perfluoropentanoic acid and 0.1% formic acid; (2) acidic positive ion (optimized for hydrophobic compounds)—extract gradient eluted from C18 (Waters UPLC BEH C18–2.1 × 100 mm, 1.7 μm) with methanol, acetonitrile, water, 0.05% perfluoropentanoic acid, and 0.01% formic acid; (3) basic negative ion—extract gradient eluted from a separate dedicated C18 column using methanol and water containing 6.5 mM ammonium bicarbonate at pH 8; and (4) negative ionization—extract gradient eluted from a HILIC column (Waters UPLC BEH Amide 2.1 × 150 mm, 1.7 μm) using water and acetonitrile with 10 mM ammonium formate at pH 10.8. In the MS analysis, the scan range varied between methods but covered the range of 70–1000 m/z.

The raw data were extracted using Metabolon's hardware and software. Compound’s identification was conducted by comparison of peaks to library entries of purified standards based on retention index, with an accurate mass match to the library of ± 10 ppm, and MS/MS forward and reverse scores between the experimental data and authentic standards. The data was manually curated. The resulted metabolic data was normalized to correct variations resulting from inter-day tuning differences in the instrument. Each compound was corrected in a run-day. The metabolomics data is provided in Supplementary Table 1.

### Cancer cell mutational profiles and gene expression data

The mutation profiles were obtained for each selected TNBC cell line from depmap (Dependency Map) portal [37]. For all selected TNBC cell lines also the gene expression data set of 190 genes across MYC, Notch, Nrf2, PI3K, Wnt and p53 pathways was obtained from Cell Model Passports portal [38]. The gene expression vales were obtained in form of Fragments Per Kilobase of transcript per Million mapped reads (FPKM).

### Statistical data analysis

The statistical data analysis was conducted using MetaboAnalyst 5.0 (https://www.metaboanalyst.ca/home.xhtml), a web server designed for comprehensive metabolomic data analysis, visualization and interpretation [39]. The normalized per run day metabolite intensities were further normalized by the sample protein content and submitted for analysis. The missing values were imputed by the min and the data was log scaled. The log scaled metabolite intensities were analyzed using parametric test. The metabolite differences with false discovery rate (FDR) adjusted p-value ≤ 0.01 and fold changes (FC) ≥ 1.5 or ≤ − 1.5 were considered significant. The data was visualized with using principle component analysis (PCA) and partial least squares discriminant analysis (PLS-DA) score plots as well as volcano plot generated with MetaboAnalyst 5.0.

A Venn diagrams were created to identify overlapping metabolites across TNBC cell lines metabolic phenotypes as well as for identification of overlapping mutated genes in those cellsusing an online tool: http://bioinformatics.psb.ugent.be/webtools/Venn.

The pathway enrichment analysis was conducted using MetaboAnalyst 5.0; The Small Molecule Pathway Database (SMPDB) containing 99 metabolite sets, based on normal human metabolic pathways, was used as referenced library.

Hierarchical clustering (HCL) was performed using the MultiExperiment Viewer (MeV) v. 4.9.0 software [40], based on FPKM, Pearson correlation as a distance measure, and average linkage clustering.

## Results

### Metabolic dysregulations in triple negative breast cancer cell lines

**Fig. 1:**
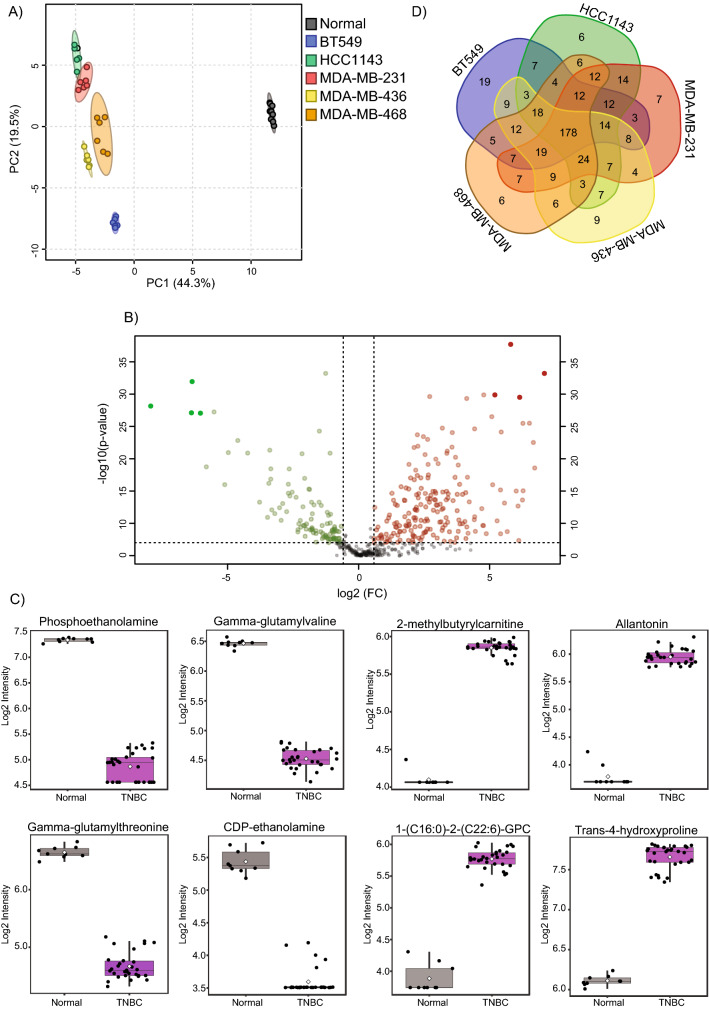
Metabolic signatures of TNBC cell lines. **A** PCA analysis reveals metabolic differences between normal and TNBC cell lines. **B** Volcano plot of metabolic features that significantly (with FDR p-value < 0.01 and fold-change ≥ 1.5 or ≤ −1.5) differ between normal and TNBC cell lines. **C** Box plots of metabolic features that show the strongest differences between normal and TNBC cell lines. **D** Venn diagram of similarities and differences across TNBC cell lines relative to normal cells

We investigated metabolic differences between 5 triple negative breast cancer cell lines (BT549, HCC1143, MDA-MB-231, MDA-MB-436, and MDA-MB-468) and normal breast cell line hTERT-HME1 using untargeted broad metabolic profiling. A total of 479 metabolites were quantified across eight primary pathways related to metabolism of amino acids, carbohydrates, cofactors and vitamins, energy, lipids, nucleotides, peptides, and xenobiotics in the samples. The clear separation, which can be seen between TNBC and control cells, on the principal component analysis (PCA) score plots (Supplementary Fig. 1, Fig. 1A), suggest strong metabolic differences between normal and cancer cells.

The separation between different cancer cell lines is also confirming cancer cell line specific metabolic fingerprints. Out of 479 detected metabolites 291 showed significant, FDR adjusted (FDR < 0.01) and simultaneously fold-change (FC) > 1.5 or ≤ 1.5 differences between normal and TNBC cells across various pathways (Supplementary Table 1). The molecules predominantly involved in the metabolism of lipids (117 molecules), amino acids (94 molecules), nucleotides (21 molecules), carbohydrates (18 molecules), and cofactors and vitamins (16 molecules) differentiate the TNBC cell lines from normal cells. Among lipids that were significantly differently regulated we found mainly glycerophospholipids (lysophosphatidylcholines, phosphatidylcholines, and phosphatidylethanolamines), sphingolipids and fatty acids; the amino acids (identified as significantly altered), including branched chain amino acids (BCAA), aromatic amino acids (AAA), methionine and glutathione metabolites. The volcano plot (Fig. 1B) highlights metabolites with FDR p-value ≤ 0.01 and fold-change (FC) ≥ 1.5 or ≤ −1.5. The top 4 hits showing up or down regulation in TNBC cells were selected for visualization (Fig. 1C).

Next, to identify similarities across cancer cell lines as well as their unique metabolic fingerprints we investigated the differences between each cancer cell line and the normal cell line. This comparison revealed 178 metabolites showing common alterations across all examined cancer cell lines as well as unique, cancer cell line specific metabolic features (Fig. 1D). The largest number of unique metabolites differentiating normal and breast cancer cell lines was identified for BT549 cell line, which is in accordance with PCA showing greatest separation of this cell line on the score plot. This data provides an overview on metabolic features differentiating TNBC cell lines from normal cells as well as emphasizes cancer cell line metabolic individuality.

### Triple negative breast cancer cell lines with low and high metastatic potential exhibit different metabolic profiles

A previous study defining metastatic potential of 500 different human cancer cell lines in vivo characterized BT549 and HCC1143 as cell lines with low metastatic potential, whereas MDA-MB-231, MDA-MB-436 and MDA-MB-468 were shown to constitute cell lines with high metastatic potential [32]. Thus, we followed this categorization and investigated whether those cell lines possess diverse metabolic program in vitro.

First, we monitored the abundance of protein markers of EMT (E-cadherin, N-cadherin, and vimentin) [21] and other proteins which were previously characterized as key components of metastatic cascade as well as molecules supporting metastasis formation (MMP2, TWIST2, p53, p38, pAkt/Akt, p-Erk1/2 and Jagged1 (JAG1)) [41–46]

**Fig. 2:**
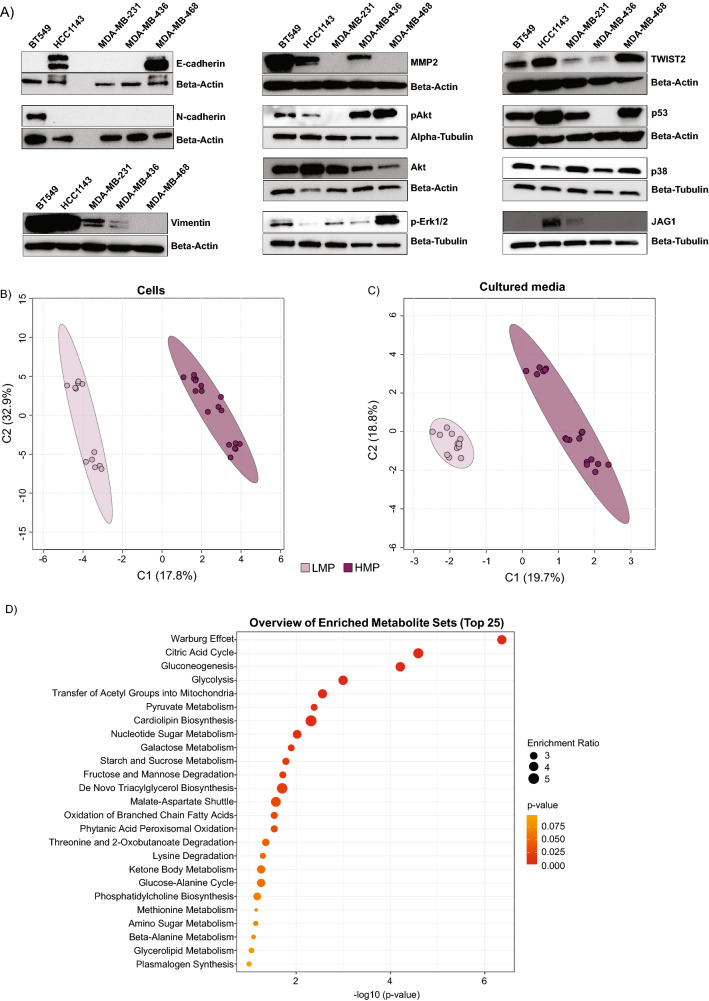
The LMP and HMP cell lines differ metabolically, and this is independent of the level of canonical markers of EMT. **A** Protein levels of EMT markers and other molecules involved in metastatic cascade. **B** and **C** partial least squares discriminant analysis (PLS-DA) score plot conducted on metabolites detected in cells and growth media. **D** Pathway enrichment analysis plot of cellular metabolism

We detected E-cadherin, an important in maintaining epithelial phenotype, in 2 cell lines including HCC1143 and MDA-MB-468 (Fig. 2A). N-cadherin, was detected only in BT549 cell line and vimentin, in four (BT549, HCC1143, MDA-MB-231, and MDA-MB-436) out of five examined cell lines (Fig. 2A). MMP2, was detected in BT549, HCC1143 and MDA-MB-436 and TWIST2 showed the highest expression in MDA-MB-468 (Fig. 2A). The cell line MDA-MB-436 was lacking expression of p53. The levels of p38 were similar across the cell lines. The cell line MDA-MB-468 showed highest levels of pAkt and p-Erk1/2. Only two cell lines namely HCC1143 and MDA-MB-231 showed expression of JAG1. This data show that selected cell lines strongly differ in canonical EMT markers as well as components involved in metastatic cascade, and those differences were not reflecting on their metastatic potential.

We have investigated mutational profiles of cancer cell lines and found unique profile for each cancer cell line (Supplementary Table 2 and Supplementary Fig. 2A). The p53 was the only molecule, which overlapped across all the investigated cell lines (Supplementary Fig. 2). Among the cell lines with HMP, we have identified 3 overlapping molecules namely Inter-alpha-trypsin inhibitor heavy chain 5 (ITIH5), semaphorin 6D (SEMA6D), and joining chain of multimeric IgA and IgM (JCHAIN). We also conducted bidirectional hierarchical cluster analysis on expression profiles of 190 genes across MYC, Notch, Nrf2, PI3K, Wnt and p53 pathways (Supplementary Table 3), known to be involved in oncogenic signaling in breast cancer [47]. The cell lines were not clustered into LMP and HMP based on the expression of selected genes (Supplementary Fig. 2 B).

Next, we investigated whether the selected cell lines displaying different metastatic potential in vivo exhibit already distinct metabolic phenotypes in vitro. To that end, we conducted metabolic profiling of both cells as well as growth media. The partial least squares discriminant analysis (PLS-DA) on metabolite profiles from both the cells as well as growth media of all TNBC cell lines revealed separation between LMP and HMP in cells (Fig. 2B) and growth media (Fig. 2C), which suggest metabolic differences between LMP and HMP. We further tested for the metabolites showing FDR (p-value ≤ 0.01) significant differences and the FC ≥ 1.5 or ≤ − 1.5 between LMP and HMP. We 92 metabolites in cells (Table 1) and 22 metabolites in growth media (Table 2), distinguishing LMP from HMP.

**Table 1:** List of cellular metabolites significantly differentiating cell lines harboring HMP from LMP

Metabolite	Pathway	Sub-pathway	FC	FDR
*N*-acetylalanine	Amino Acid	Alanine Metabolism	1.58	5.57 × 10^–6^
Gamma-carboxyglutamate		Glutamate Metabolism	2.71	3.04 × 10^–9^
4-Hydroxyglutamate			4.50	9.47 × 10^–4^
Beta-citrylglutamate			5.51	3.38 × 10^–4^
Glutamate gamma-methyl ester			5.54	4.05 × 10^–3^
Cysteinylglycine		Glutathione Metabolism	1.99	4.93 × 10^–4^
S-lactoylglutathione			5.58	4.03 × 10^–4^
*N*-acetylglycine		Glycine Metabolism	2.66	5.06 × 10^–3^
4-Imidazoleacetate		Histidine Metabolism	5.18	1.87 × 10^–3^
3-Methyl-2-oxovalerate		BCAA Metabolism	2.16	2.36 × 10^–3^
4-Methyl-2-oxopentanoate			2.77	1.71 × 10^–3^
3-Methyl-2-oxobutyrate			4.64	9.40 × 10^–6^
Lanthionine		Methionine Metabolism	0.34	1.77 × 10^–3^
Methionine sulfoxide			0.59	1.18 × 10^–4^
*N*-formylmethionine			1.74	1.09 × 10^–5^
Spermidine		Polyamine Metabolism	0.42	8.56 × 10^–11^
5-Methylthioadenosine (MTA)			1.63	3.54 × 10^–3^
Kynurenine		Tryptophan Metabolism	0.10	4.33 × 10^–6^
Serotonin			2.48	3.48 × 10^–4^
*O*-methyltyrosine		Tyrosine Metabolism	0.65	5.70 × 10^–6^
Citrulline		Urea cycle metabolism	0.58	6.17 × 10^–4^
*N*-acetylglucosamine/*N*-acetylgalactosamine	Carbohydrate	Aminosugar Metabolism	0.49	6.06 × 10^–4^
Erythronate			2.98	9.05 × 10^–6^
Fructose		Fructose & Mannose Metabolism	2.97	9.25 × 10^–8^
Mannose-6-phosphate			7.36	3.04 × 10^–9^
3-Phosphoglycerate		Glycolysis/Gluconeogenesis	2.04	1.09 × 10^–5^
Phosphoenolpyruvate			2.06	1.96 × 10^–5^
Fructose 1,6-2D/glucose 1,6-2P/myo-inositol-2P			2.29	5.71 × 10^–3^
2-phosphoglycerate			2.97	3.04 × 10^–9^
Glucose 6-phosphate			4.16	2.39 × 10^–9^
Fructose-6-phosphate			6.68	3.97 × 10^–6^
UDP-glucose		Nucleotide Sugar	1.80	2.91 × 10^–3^
UDP-galactose			2.02	1.92 × 10^–3^
Ribonate		Pentose Phosphate	2.63	1.77 × 10^–3^
6-phosphogluconate			5.22	4.27 × 10^–8^
Nicotinamide adenine dinucleotide	Cofactors and Vitamins	Nicotinamide Metabolism	1.80	2.51 × 10^–4^
Nicotinamide adenine dinucleotide R			1.89	1.16 × 10^–3^
Phosphopantetheine		Pantothenate Metabolism	0.32	1.56 × 10^–3^
Coenzyme A			1.78	3.59 × 10^–4^
Thiamin (Vitamin B1)		Thiamine Metabolism	1.84	3.21 × 10^–4^
Thiamin diphosphate			3.64	9.05 × 10^–6^
Citrate	Energy	TCA Cycle	1.54	8.28 × 10^–11^
Aconitate [cis or trans]			1.73	2.08 × 10^–6^
Malate			1.79	1.80 × 10^–5^
Isocitrate			1.89	4.01 × 10^–3^
Alpha-ketoglutarate			3.03	9.25 × 10^–8^
2-methylcitrate/homocitrate			4.03	3.69 × 10^–7^
Succinylcarnitine			9.09	1.45 × 10^–7^
Ceramide (d18:1/14:0 d16:1/16:0)	Lipid	Ceramides	0.43	5.73 × 10^–3^
Myristoyl dihydrosphingomyelin		Dihydrosphingomyelins	0.21	6.38 × 10^–4^
*N*-linoleoyltaurine		Endocannabinoid	0.55	2.83 × 10^–3^
Palmitoyl ethanolamide			0.48	7.61 × 10^–3^
Propionylcarnitine		Fatty Acid Metabolism (Acyl Carnitine)	7.88	1.10 × 10^–4^
Acetylcarnitine			2.90	5.37 × 10^–7^
Linoleoylcarnitine (C18:2)			0.15	9.40 × 10^–6^
Myristoleoylcarnitine (C14:1)			0.37	1.43 × 10^–5^
Oleoylcarnitine (C18:1)			0.20	2.36 × 10^–3^
Palmitoleoylcarnitine (C16:1)			0.23	3.80 × 10^–03^
Glycerophosphoglycerol		Glycolipid Metabolism	4.64	9.05 × 10^–6^
Galactosylglycerol			4.61	3.76 × 10^–3^
1-Linoleoyl-GPE (18:2)		Lysophospholipid	4.83	1.15 × 10^–6^
2-Stearoyl-GPE (18:0)			3.72	1.86 × 10^–6^
1-Linoleoyl-GPC (18:2)			1.94	2.98 × 10^–6^
1-Stearoyl-GPE (18:0)			2.64	4.64 × 10^–6^
1-Oleoyl-GPE (18:1)			1.79	1.64 × 10^–4^
1-Stearoyl-GPI (18:0)			0.48	6.06 × 10^–4^
1-Stearoyl-GPC (18:0)			5.90	2.91 × 10^–3^
1-Palmitoyl-GPI (16:0)			0.55	2.95 × 10^–3^
1-Arachidonoyl-GPE (20:4n6)			1.62	7.69 × 10^–3^
1-Stearoyl-GPG (18:0)			2.34	8.40 × 10^–3^
1-Oleoylglycerol (18:1)		Monoacylglycerol	0.10	5.33 × 10^–3^
1-Myristoyl-2-arachidonoyl-GPC (14:0/20:4)		Phosphatidylcholine	0.49	3.16 × 10^–4^
1-Stearoyl-2-oleoyl-GPG (18:0/18:1)		Phosphatidylglycerol	2.91	1.64 × 10^–5^
Choline phosphate		Phospholipid Metabolism	1.90	4.90 × 10^–5^
Choline			0.62	1.77 × 10^–3^
Sphingadienine		Sphingolipid Synthesis	2.39	7.73 × 10^–7^
Phytosphingosine			1.90	8.40 × 10^–4^
Sphingosine		Sphingosines	1.97	2.33 × 10^–7^
AICA ribonucleotide	Nucleotide	Purine Metabolism	18.26	4.36 × 10^–5^
2′-deoxyadenosine 5′-triphosphate			2.93	3.90 × 10^–4^
2′-deoxyadenosine 5′-diphosphate			5.67	1.51 × 10^–7^
Guanosine 5′-triphosphate			1.53	4.36 × 10^–5^
Guanosine 5′- diphosphate			1.79	5.65 × 10^–6^
Guanine			4.17	5.80 × 10^–3^
Cytidine 2′3′-cyclic monophosphate		Pyrimidine Metabolism	0.09	4.41 × 10^–11^
Cytidine 5′-monophosphate			2.89	3.42 × 10^–3^
Orotate			1.59	9.34 × 10^–4^
Dihydroorotate			1.64	7.65 × 10^–3^
Orotidine			4.93	9.63 × 10^–4^
3-Ureidopropionate			2.48	1.90 × 10^–5^
4-Methylbenzenesulfonate	Xenobiotics	Chemical	0.27	7.16 × 10^–3^
Gluconate		Food Component/Plant	3.60	2.43 × 10^–4^

Times symbol indicate nicotinamide adenine dinucleotide reduced (NADH)

**Table 2:** List of growth medium metabolites significantly differentiating cell lines harboring HMP from LMP

Metabolite	Pathway	Sub-pathway	FC	FDR
Guanidinoacetate	Amino Acid	Creatine Metabolism	0.39	1.35 × 10^–3^
2-Hydroxy-3-methylvalerate		BCAA Metabolism	3.62	1.19 × 10^–13^
Alpha-hydroxyisocaproate			4.08	2.30 × 10^–6^
1-Carboxyethylleucine			0.66	9.39 × 10^–5^
4-Methyl-2-oxopentanoate			2.77	4.91 × 10^–4^
3-Methyl-2-oxobutyrate			2.19	5.88 × 10^–4^
3-Methyl-2-oxovalerate			2.44	6.46 × 10^–4^
Ethylmalonate			0.49	2.89 × 10^–3^
Cystathionine		Methionine Metabolism	0.57	1.92 × 10^–3^
Kynurenine		Tryptophan Metabolism	0.12	1.51 × 10^–5^
3-Hydroxyhexanoate	Lipid	Fatty Acid, Monohydroxy	2.67	7.38 × 10^–3^
5-Dodecenoate (12:1n7)		Medium Chain Fatty Acid	0.31	2.61 × 10^–5^
Choline		Phospholipid Metabolism	0.48	7.05 × 10^–4^
Docosahexaenoate (22:6n3)		Polyunsaturated Fatty Acid (n3 and n6)	0.34	2.10 × 10^–7^
Eicosapentaenoate (20:5n3)			0.52	4.35 × 10^–3^
Thymine	Nucleotide	Pyrimidine Metabolism	0.33	9.39 × 10^–5^
Uracil			0.49	1.10 × 10^–5^
3-Ureidopropionate			2.39	1.09 × 10^–4^
Phenylacetylglycine	Peptide	Acetylated Peptides	0.54	9.80 × 10^–3^
Gamma-glutamylglycine		Gamma-glutamyl Amino Acid	1.67	5.53 × 10^–3^
p-Aminobenzoate	Xenobiotics	Benzoate Metabolism	0.45	3.50 × 10^–3^
3-hydroxyhippurate			1.72	6.75 × 10^–3^

Among the 92 cellular metabolites that are significantly different between HMP and LMP cell lines, we found 30 lipids (fatty acids and lysophospholipids), 21 amino acids involved in glutamate, BCAA and methionine metabolism, 14 carbohydrates contributing mainly to glycolysis, 12 nucleotides, 7 TCA cycle metabolites, 6 cofactors and vitamins, and 2 xenobiotics. We conducted enrichment analysis on cellular metabolites using MetaboAnalyst 5.0, and found FDR significant enrichment (p-value < 0.05) in Warburg effect, TCA cycle, gluconeogenesis and glycolysis (Fig. 2D).

Among 22 metabolites measured in growth media that show significant differences between HMP and LMP cell lines 10 were amino acids including 7 molecules involved in BCAA metabolism, 5 lipids, 3 nucleotides and 2 xenobiotics. The metabolic signatures of HMP cell lines identified in media were not showing significant any enrichment. Taken together, these findings indicate that cell lines with LMP and HMP differ metabolically in vitro, and those differences are independent of the EMT markers.

#### Metabolic pathways contributing to metastatic potential of cancer cells

We constructed the metabolic pathway based on the molecules showing significant differences between HMP and LMP cell lines (Supplementary Fig. 3). The metabolic signatures differentiating HMP from LMP cell lines focuses around three main pathways namely glycolysis, TCA cycle and lipid metabolism. In addition, we identified a significant increase in the levels of 2-hydroxy-3-methylvalerate (p-value = 1.19 × 10^–13^; FC = 3.62), alpha-hydroxyisocaproate (p-value = 2.30 × 10^–6^; FC = 4.08) in growth media and 4-methyl-2-oxopentanoate (media: p-value = 4.91 × 10^–4^; FC = 2.77; cell: p-value = 1.71 × 10^–3^; FC = 2.77), 3-methyl-2-oxobutyrate (media: p-value = 5.88 × 10^–4^; FC = 2.19; cell: p-value = 9.40 × 10^–6^; FC = 4.64), and 3-methyl-2-oxovalerate (media: p-value = 6.46 × 10^–4^; FC = 2.44; cell: p-value = 2.36 × 10^–3^; FC = 2.16) in both growth media and cells (Supplementary Fig. 4). All those mentioned metabolites are products of BCAA catabolic pathway; the largest differences observed in the levels of 2-hydroxy-3-methylvalerate, alpha-hydroxyisocaproate in media and 3-methyl-2-oxobutyrate indicate accelerated catabolism of isoleucine, leucine and valine, respectively. Elevated levels of those metabolites in growth media suggest their release by the HMP cells.

In HMP cells, we also observed the elevated levels of cellular gamma-carboxyglutamate (p-value = 3.04 × 10^–9^; FC = 2.71) and 4-hydroxyglutamate (p-value = 9.47 × 10^–4^; FC = 4.50), which are products of glutamate metabolism; the glutamate and glutamine levels were not different between cell lines with distinct metastatic potential (Supplementary Fig. 5A). Similarly, differences in the levels of products of tryptophane metabolism, namely kynurenine in both media (p-value = 9.47 × 10^–4^; FC = 4.50) and cells (p-value = 9.47 × 10^–4^; FC = 4.50) as well as cellular serotonin level (p-value = 9.47 × 10^–4^; FC = 4.50) but not tryptophan, were found significantly different between HMP and LMP (Supplementary Fig. 5B). Additionally, lower cellular level of spermidine (p-value = 8.56 × 10^–11^; FC = 0.42) was found in HMP in comparison to LMP without changes in other polyamines (Supplementary Fig. 5C).

#### Elevated glycolysis is a signature of cell lines harboring high metastatic potential

**Fig. 3:**
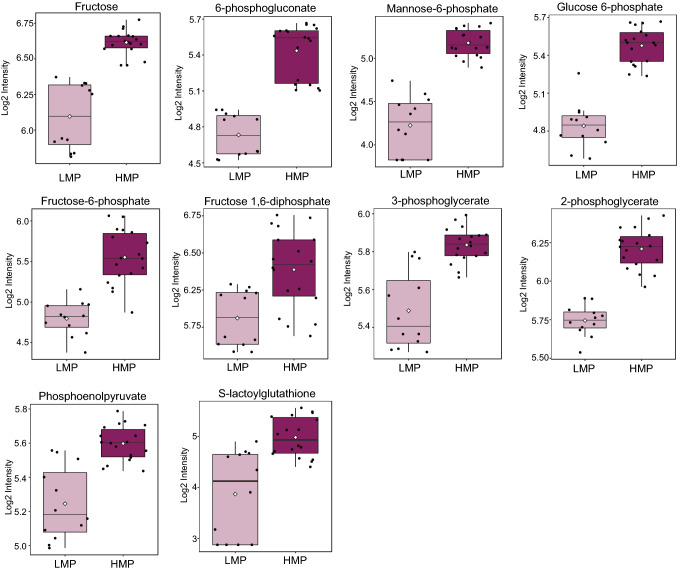
The cell lines with HMP manifest increased glycolysis. The data is presented in the form of box plots. Light purple indicates cells with LMP; dark purple indicates cells with HMP. (Color figure online)

Elevated glucose metabolism is a well-known hallmark of cancer [48], which was shown to be even more dysregulated in metastatic cells [49]. We found enrichment in glucose metabolism across metabolites differentiating cell lines with HMP and LMP (Fig. 2D). We further focused on the molecules involved in carbohydrate metabolism showing significant differences between HMP and LMP cell lines (Fig. 3).

The molecules involved in glycolysis including glucose 6-phosphate, fructose-6-phosphate, fructose 1,6-diphosphate, 3-phosphoglycerate, 2-phosphoglycerate, and phosphoenolpyruvate (PEP) (Fig. 3, and Table 1) were all elevated in cell lines harboring HMP. The levels of lactate and pyruvate were not significantly different between cell lines with distinct metastatic potential, but the level of S-lactoylglutathione, which is a part of pyruvate pathway and can be metabolized into lactate, was significantly elevated in HMP cell lines. The glycolytic pathway is interconnected with pentose phosphate metabolism as well as contributes to purine and pyrimidine synthesis [50]. We observed elevated levels in HMP of two molecules (6-phosphogluconate and ribonate) of pentose phosphate pathway (Fig. 3, and Table 1) as well as elevated levels of purines containing adenine (2'-deoxyadenosine 5'-diphosphate (p-value = 1.51 × 10^–7^; FC = 5.67) and 2′-deoxyadenosine 5′-triphosphate (p-value = 3.90 × 10^–4^; FC = 2.93)) and guanine (guanine (p-value = 5.80 × 10^–3^; FC = 4.17), guanosine 5′-diphosphate (p-value = 5.65 × 10^–6^; FC = 1.79) and guanosine 5′-triphosphate (p-value = 4.36 × 10^–5^; FC = 1.53)) and AICA ribonucleotide (p-value = 4.36 × 10^–5^; FC = 18.26) as well as pyrimidines containing orotate (dihydroorotate (p-value = 7.65 × 10^–3^; FC = 1.64) and orotate (p-value = 9.34 × 10^–4^; FC = 1.59)) and cytidine (cytidine 5′-monophosphate (p-value = 3.42 × 10^–3^; FC = 2.89)). The level of cytidine 2′3′-cyclic monophosphate was significantly (p-value = 4.41 × 10^–11^; FC = 0.09) decreased in cell lines with HMP in comparison with those of LMP. The HMP cell lines displayed elevated levels of fructose and manose-6-phospahete in comparison with LMP cell lines (Fig. 3, and Table 1). Thus, it could be reasoned that cell lines harboring HMP exhibit elevated glycolysis, potentially to supply nucleotide synthesis.

#### Upregulated citrate metabolism but not entire TCA cycle is a hallmark of cell lines with HMP

**Fig. 4:**
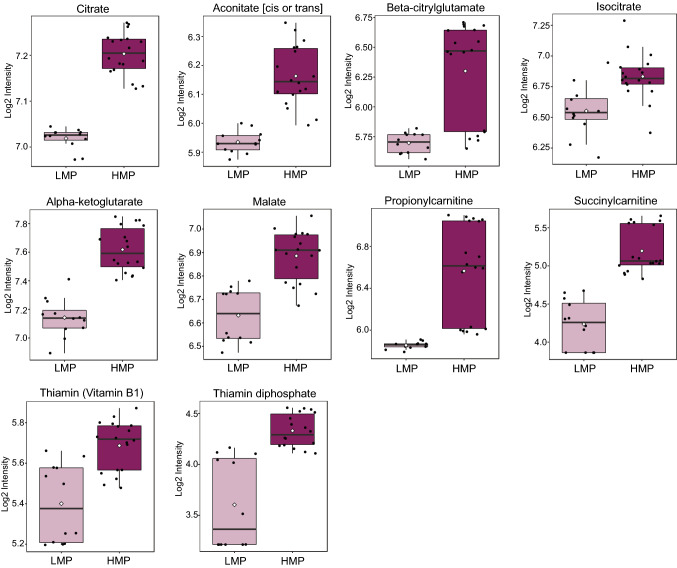
The cell lines harboring HMP exhibit enhanced citrate metabolism. The data is presented in the form of box PLOTS. Light purple indicates cells with LMP; dark purple indicates cells with HMP. (Color figure online)

Dysregulated metabolism of TCA cycle molecules, including succinate, fumarate, alpha-ketoglutarate, 2-hydroxyglutarate and citrate, was previously attributed to metastatic cells [23]. Our enrichment analysis suggested that TCA cycle metabolism is enhanced in TNBC cell lines with HMP (Fig. 2D). We observed that mainly citrate and the components of citrate metabolism, including aconitate [cis or trans], beta-citrylglutamate, isocitrate and alpha-ketoglutarate, were significantly elevated in cell lines harboring HMP (Fig. 4 and Table 1). The levels of succinyl-CoA, succinate and fumarate were not significantly different between HMP and LMP cell lines. The malate level was higher in HMP in comparison to LMP cell lines. We have also found increased levels of succinyl carnitine and propionyl carnitine (Fig. 4 and Table 1) which can contribute to TCA cycle on the succinyl-CoA level. Interestingly, levels of thiamine and thiamine diphosphate, which are critical for the activity of TCA cycle enzymes including pyruvate dehydrogenase (PDH) and alpha-ketoglutarate dehydrogenase (α- KGDH) [51], were significantly elevated in HMP cell lines (Fig. 4 and Table 1). Taken together, the citrate metabolism, rather than entire TCA cycle, is upregulated in cell lines with HMP.

#### Dysregulated lipid metabolism is a feature of cell lines with HMP

**Fig. 5:**
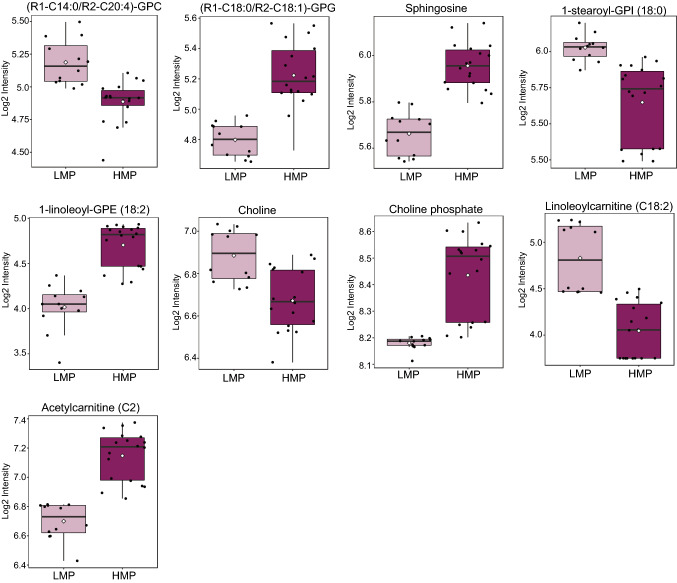
The components of lipid metabolism differentiate HMP from LMP cell lines. Box plots showing examples of alterations in molecules involved in various lipid metabolism pathways. Light purple indicates cells with LMP; Dark purple indicate cells with HMP. (Color figure online)

We identified 30 different molecules involved in lipid metabolism, which significantly differentiate HMP from LMP cell lines (Table 1). To provide further insight regarding their potential contribution to cancer cell metastatic we have analyzed them in the context of a pathway (Supplementary Fig. 3). The levels of lysophospholipids were differently regulated between LMP and HMP namely lysophosphatidylinositols were significantly decreased whereas lysophosphatidylcholines and lysophosphatidylethanolamines were significantly elevated in cell lines with HMP in comparison to the one with LMP (Fig. 5 and Supplementary Fig. 3). The levels of choline were decreased whereas phosphocholine levels were elevated in HMP cell lines (Fig. 5). Out of 43 measured glycerophospholipids only two (including elevation in HMP 1-stearoyl-2-oleoyl-GPG (18:0/18:1) and decrease in HMP 1-myristoyl-2-arachidonoyl-GPC (14:0/20:4)) were showing significant differences between HMP and LMP cell lines (Fig. 5). We did not observe any differences in the free fatty acid levels between LMP and HMP but found significantly lower levels of four acylcarnitines (linoleoylcarnitine (C18:2), oleoylcarnitine (C18:1), palmitoleoylcarnitine (C16:1), and myristoleoylcarnitine (C14:1)), in cell lines harboring HMP (Fig. 5 and Table 1). The acylcarnitines are required to transport free fatty acids across mitochondrial membrane for beta-oxidation. The lower level of acylcarnitines could suggest decrease in beta-oxidation in HMP cell lines and incorporation of fatty acids into lysophospholipids which were significantly elevated (Fig. 5 and Table 1). The level of acetylcarnitine was significantly elevated in HMP cell lines. We also identified alteration in sphingolipid metabolism; the levels of sphingosine and phytosphingosine were elevated whereas sphingomyelins decreased in the cell lines with HMP. Taken together, this data indicates that cancer cell lines harboring distinct metastatic potential activate different programs of lipid metabolism. The lipid dysregulation in HMP manifests in increased levels of glycerophospholipids and acetylcarnitine and decreased levels of acylcarnitines further suggesting potential enhancement in lipid synthesis.

## Discussion

Dysregulated metabolism plays a vital role in cancer cell progression and metastasis [22, 23, 25, 26, 52–56]. In this study, we have shown that TNBC cell lines differentiating in their metastatic potential in vivo exhibit different metabolic profile already in vitro, and those differences are independent of the abundance of canonical EMT markers. The cell line MDA-MB-468 reported as with HMP in vivo, was not expressing any of the canonical markers of EMT. Nevertheless, to undergo EMT this cell line requires exposure to epidermal growth factor (EGF) [57], thus in vitro abundance of EMT markers was not expected for this particular cell line. The analysis of mutational profiles revealed 3 molecules namely ITIH5, SEMA6D, and JCHAIN overlapping across the cell lines with HMP. Interestingly, ITIH5 and SEMA6D were reported as contributors to cancer cell metastasis [58, 59].

Cell lines harboring HMP displayed enrichment in glycolysis and TCA cycle, as well as dysregulated metabolism of lipids. The elevated levels of products of BCAA catabolism in cells as well as growth media suggested accelerated BCAA catabolism in TNBC cell lines with HMP. Additionally, we found significant differences in the levels of gamma-carboxyglutamate, 4-hydroxyglutamate, kynurenine, serotonin, and spermidine between HMP and LMP cell lines.

Elevated glycolysis was previously described as a feature of metastatic cells, which supports cancer cell survival under energy deficient conditions occurring after cell dissociation from the primary tumor and establishment of metastatic niche [54]. Moreover, enhanced glycolysis was shown to support release of exosome by metastasizing cells, which is a crucial step in the metastatic cascade, and thus linking dysregulated glucose metabolism with cancer cell ability to extravasate at distant premetastatic niche [55]. Therefore, the enhanced glycolytic program in the cells with HMP, observed in our study, is in concordance with previous reports and further pinpoints metabolic advantage of HMP over LMP cell lines. Elevated glycolysis in cancer cells frequently results in accumulation of methylglyoxal, which is further metabolized to S-lactoylglutathione by glyoxalase 1 (GLO1) and subsequently to lactate in the reaction catalyzed by glyoxalase 2 (GLO2) [60]. The elevated expression of GLO1 was found in basal TNBC and was shown to be essential for the survival of breast cancer stem cells [61]. The elevated level of S-lactoylglutathione observed in our study in cell lines with HMP further underscores enhanced glycolytic program in those cells as well as suggests increased expression of GLO1. Importantly, S-lactoylglutathione could serve as reservoir of lactate, shown to be a key player during metastasis by stimulating angiogenesis and increasing extracellular acidification to evade the immune system [62], which could be considered as further metabolic advantages of HMP cell lines. Moreover, significantly elevated levels of S‑lactoylglutathione were found in subjects suffering of gastric cancer resistant to neoadjuvant chemotherapy, and S‑lactoylglutathione was suggested as a metabolic marker differentiating chemo-sensitive from chemo-resistant subjects [63].

Elevated glycolysis in cancer cells is frequently linked with enhanced lactate synthesis, however differences in the lactate level between cell lines harboring HMP and LMP was not found. Nevertheless, we found increased level of citrate and molecules involved in citrate metabolism, which could suggest higher glucose contribution to TCA cycle in HMP cell lines. The greater flux of glucose into TCA cycle was recently reported in metastatic colorectal cancer cell lines [56]. The enhanced glycolysis along with intracellular accumulation of citrate were shown to enhance TNBC cell invasion and metastasis via AKT/ERK signaling pathway [53]. In our study in addition to elevated citrate level we found increased levels of other molecules involved in citrate metabolism including aconitate [cis or trans], isocitrate, alpha-ketoglutarate beta-citrylglutamate as well as succinylcarnitine further highlighting potential importance of this pathway in governing the metastatic cascade. Wu et al. reported elevated serum succinate levels along with expression of succinate receptor 1 (SUCNR1) in lung cancer patients as key factors involved in modulation of tumor microenvironment potentially promoting metastasis [64]. Furthermore, beta-citrylglutamate was shown as an activator of aconitase, which catalyzes isocitrate formation [65]. These results indicate that metabolism of citrate rather than dysregulation of the entire TCA cycle predispose metastatic potential in TNBC cell lines. Importantly, the elevated citrate level is an indicator of energy excess and cell readiness for fatty acid synthesis. Citrate is cleaved by ATP citrate lyase and the achieved oxaloacetate reenters into TCA cycle in the form of malate [66]. The elevated citrate metabolism along with increased malate level in HMP cell lines further suggests enhanced fatty acid and other lipid synthesis in those cells. In concordance, we have observed decrease in the acylcarnitine levels, which are indicators of fatty acid catabolism, as well as an increase in glycerophospholipid levels in HMP cell lines. This observation suggests that enhanced citrate metabolism contributes to accelerated lipid synthesis in HMP cell lines, which was previously linked with tumor progression [67]. Noteworthy, strong association between brain metastasis and enhanced lipid metabolism was reported by Jin et al. who showed increased levels of cholesterol species, phosphatidylcholines and sphingomyelins and decreased triacylglycerol levels in highly brain metastatic cells [32]. Our study is in agreement with his observation as we found elevated levels of glycerophospholipids and sphingomyelins in HMP cell lines and also suggesting enhanced lipid synthesis in cells with HMP. Changes in various lipid levels were found in subjects with non–small cell lung cancer (NSCLC) after tumor resection, further suggested role of lipid metabolism in tumor progression [68] and pointing it as a treatment target [69].Additionally, we observed an increase in the catabolic pathway of BCAA in HMP cell lines manifested by the accumulation of their products of catabolism in both cell and growth media. The products of leucine catabolism, namely 2-hydroxy-3-methylvalerateand alpha-hydroxyisocaproate, displayed the greatest accumulation in HMP cell lines in comparison with LMP cell lines. The increased BCAA catabolism could contribute to enhanced energy generation and biomass production as well as promotion of mTOR signaling, which is a known cancer cell molecular pathway [70]. Furthermore, it was shown that inhibition of leucine uptake suppresses mTOR signaling and promotes apoptosis in breast cancer cell lines [71]. Moreover, the enhanced activity of branched-chain α-keto acid dehydrogenase kinase (BCKDK), the key enzyme of BCAAs metabolism, was shown to promote migration, invasion and EMT of colorectal cancer [72]. Therefore, it could be reasoned that increased BCAA catabolism observed in HMP cell lines contribute to their metabolic advantage which empowers their metastatic potential.

Noteworthy, the levels of thiamine and thiamine diphosphate, which are critical for activity of enzymes involved in TCA cycle, pentose phosphate and BCAA metabolism, were elevated in cell lines with HMP further underscoring enhanced metabolic potential in those cell lines. The importance of thiamine in cancer cell metabolism was recently suggested [51]. Thus, it could be reasoned that HMP cell lines activate thiamine metabolism to ensure enhanced activity of TCA cycle, pentose phosphate and BCAA metabolism.

Coagulation proteins along with platelets have been shown to promote pro-survival signaling during metastasis [73]. The HMP cell lines displayed elevated levels of γ-carboxyglutamine, which is involved in coagulation cascade; the γ-carboxyglutamic acid residues play an important role in coagulation by governing the activation and binding of circulating blood-clotting enzymes to cell membrane surface [74]. Moreover, the HMP cell lines exhibited an increased level of 2'-deoxyadenosine 5'-triphosphate and 2'-deoxyadenosine 5'-diphosphate, which are molecules of adenine nucleotide metabolism. The role of adenine nucleotides in extravasation was previously suggested and linked with platelet activation by cancer cells [75]. Furthermore, 4-hydroxyglutamate which was also elevated in HMP cell lines, could potentially play a role in platelet activation as this molecule was identified as metabolic marker of preeclampsia, a health condition associated with coagulation and platelet activation [76]. Thus, the cell lines harboring HMP possess metabolic features potentially supporting coagulation and platelet activation, which are important contributors of the metastatic cascade.

## Conclusions

In conclusion, our study provides new insights into cancer metastasis from the perspective of dysregulated metabolism. The landscape of metabolic dysregulations characterized in our study could serve as a roadmap for identification of treatment strategies targeting cancer cells with enhanced metastatic potential. We identified metabolic advantages of cell lines with high metastatic potential beyond enhanced glycolysis by pinpointing the role of BCAA catabolism as well as molecules supporting coagulation and platelet activation as important contributors to metastatic cascade. A future prospective would be to probe those identified metabolic dysregulations as therapeutic targets.

## Supplementary Information

Below is the link to the electronic supplementary material.

Pairwise score plot for top five principal component (PC). Grey - hTERT-HME1; blue - BT549; green - HCC1143; read - MDA-MB-231; yellow - MDA-MB-436; orange - MDA-MB-468. Supplementary file1 (PDF 146 kb)

A) Venn diagram showing overlap of mutated molecules across the five investigated TNBC cell lines. B) Hierarchical clustering (HCL) analysis of expression profiles of 190 genes across MYC, Notch, Nrf2, PI3K, Wnt and p53 pathways in five investigated cancer cell lines.. Supplementary file2 (PDF 587 kb)

Metabolic pathway depicting metabolic differences between HMP and LMP cell lines. Red – metabolites significantly elevated in HMP in comparison with LMP; Green – metabolites significantly decreased in HMP in comparison with LMP; Grey – measured metabolites not significantly different between HMP and LMP; White – metabolite not detected. Supplementary file3 (PDF 505 kb)

. Box plots showing products of BCAA catabolism differentiating significantly HMP and LMP cell lines in A) growth medium and B) cells. Light and dark purple indicate cell lines with LMP and HMP respectively. Supplementary file4 (PDF 459 kb)

Box plots showing metabolites differentiating significantly HMP and LMP cell lines involved in A) glutamate metabolism; B) tryptophane metabolism and C) polyamine metabolism. Light and dark purple indicate cell lines with LMP and HMP respectively. Supplementary file5 (PDF 437 kb)

Metabolomics data. Supplementary file6 (XLSX 216 kb)

Mutational profiles of TNBC cell lines obtained from Dependency Map portal. Supplementary file7 (XLSX 123 kb)

Expression profiles of 190 genes across MYC, Notch, Nrf2, PI3K, Wnt and p53 pathways obtained from Cell Model Passports portal. Supplementary file8 (XLSX 20 kb)

List of metabolites showing FDR significant differences between TNBC and normal cells. Supplementary file9 (XLSX 31 kb)

## Data Availability

All the data is available along with the manuscript.

## Abbreviations

TNBC: Triple negative breast cancer
LMP: Low metastatic potential
HMP: High metastatic potential
ER: Estrogen receptor
PR: Progesterone receptor
HER2: Human epidermal growth factor receptor 2
EMT: Epithelial-mesenchymal transition
PCA: Principal component analysis
PLS-DA: Partial least squares discriminant analysis
FDR: False discovery rate
FC: Fold-change
PBS: Phosphate buffered saline
PMSF: Phenyl methane sulfonyl fluoride
UPLC: Ultra-performance liquid chromatography
MS: Mass spectrometry
ESI: Electrospray ionization
BCAA: Branched chain amino acids
TCA: Tricarboxylic acid
PEP: Phosphoenolpyruvate

## References

[1] Spano D, Heck C, De Antonellis P (2012). Molecular networks that regulate cancer metastasis. Semin Cancer Biol.

[2] Fidler IJ, Kripke ML (2015). The challenge of targeting metastasis. Cancer Metastasis Rev.

[3] Lambert AW, Pattabiraman DR, Weinberg RA (2017). Emerging biological principles of metastasis. Cell.

[4] Foulkes WD, Smith IE, Reis-Filho JS (2010). Triple-negative breast cancer. N Engl J Med.

[5] Garrido-Castro AC, Lin NU, Polyak K (2019). Insights into molecular classifications of triple-negative breast cancer: Improving patient selection for treatment. Cancer Discov.

[6] Fares J, Fares MY, Khachfe HH (2020). Molecular principles of metastasis: a hallmark of cancer revisited. Signal Transduct Target Ther.

[7] Nicholson JK, Lindon JC, Holmes E (1999). “Metabonomics”: understanding the metabolic responses of living systems to pathophysiological stimuli via multivariate statistical analysis of biological NMR spectroscopic data. Xenobiotica.

[8] Suhre K, Shin S-Y, Petersen A-K (2011). Human metabolic individuality in biomedical and pharmaceutical research. Nature.

[9] Beger RD, Dunn W, Schmidt MA (2016). Metabolomics enables precision medicine: “A White Paper, Community Perspective”. Metabolomics.

[10] Wishart DS (2016). Emerging applications of metabolomics in drug discovery and precision medicine. Nat Rev Drug Discov.

[11] Seyfried TN, Flores RE, Poff AM, D’Agostino DP (2014). Cancer as a metabolic disease: implications for novel therapeutics. Carcinogenesis.

[12] Warburg O (1925). The metabolism of carcinoma cells. J Cancer Res.

[13] Altman BJ, Stine ZE, Dang CV (2016). From Krebs to clinic: glutamine metabolism to cancer therapy. Nat Rev Cancer.

[14] Koundouros N, Poulogiannis G (2020). Reprogramming of fatty acid metabolism in cancer. Br J Cancer.

[15] Bueno MJ, Jimenez-Renard V, Samino S (2019). Essentiality of fatty acid synthase in the 2D to anchorage-independent growth transition in transforming cells. Nat Commun.

[16] Villa E, Ali ES, Sahu U, Ben-Sahra I (2019). Cancer cells tune the signaling pathways to empower de novo synthesis of nucleotides. Cancers (Basel)..

[17] Gross MI, Demo SD, Dennison JB (2014). Antitumor activity of the glutaminase inhibitor CB-839 in triple-negative breast cancer. Mol Cancer Ther.

[18] Falchook G, Infante J, Arkenau HT (2021). First-in-human study of the safety, pharmacokinetics, and pharmacodynamics of first-in-class fatty acid synthase inhibitor TVB-2640 alone and with a taxane in advanced tumors. EClinicalMedicine.

[19] Halama A, Kulinski M, Dib SS (2018). Accelerated lipid catabolism and autophagy are cancer survival mechanisms under inhibited glutaminolysis. Cancer Lett.

[20] Achkar IW, Kader S, Dib SS (2020). Metabolic signatures of tumor responses to doxorubicin elucidated by metabolic profiling in ovo. Metabolites.

[21] Pera B, Krumsiek J, Assouline SE (2018). Metabolomic Profiling Reveals Cellular Reprogramming of B-Cell Lymphoma by a Lysine Deacetylase Inhibitor through the Choline Pathway. EBioMedicine.

[22] Bergers G, Fendt SM (2021). The metabolism of cancer cells during metastasis. Nat Rev Cancer.

[23] Wei Q, Qian Y, Yu J, Wong CC (2020). Metabolic rewiring in the promotion of cancer metastasis: mechanisms and therapeutic implications. Oncogene.

[24] Sun G, Zhang X, Liang J (2021). Integrated MOlecular characterization of fumarate hydratase–deficient renal cell carcinoma. Clin Cancer Res.

[25] Aspuria PJP, Lunt SY, Väremo L (2014). Succinate dehydrogenase inhibition leads to epithelial-mesenchymal transition and reprogrammed carbon metabolism. Cancer Metab.

[26] Colvin H, Nishida N, Konno M (2016). Oncometabolite D-2-hydroxyglurate directly induces epithelial-mesenchymal transition and is associated with distant metastasis in colorectal cancer. Sci Rep.

[27] Sasaki M, Knobbe CB, Munger JC (2012). IDH1(R132H) mutation increases murine haematopoietic progenitors and alters epigenetics. Nature.

[28] Their JP (2002). Epithelial-mesenchymal transitions in tumor progression. Nat Rev Cancer.

[29] Halama A, Guerrouahen BS, Pasquier J (2017). Nesting of colon and ovarian cancer cells in the endothelial niche is associated with alterations in glycan and lipid metabolism. Sci Rep.

[30] Teoh ST, Ogrodzinski MP, Ross C (2018). Sialic acid metabolism: a key player in breast cancer metastasis revealed by metabolomics. Front Oncol.

[31] Lu M, Zhu WW, Wang X (2019). ACOT12-dependent alteration of acetyl-CoA drives hepatocellular carcinoma metastasis by epigenetic induction of epithelial-mesenchymal transition. Cell Metab.

[32] Jin X, Demere Z, Nair K (2020). A metastasis map of human cancer cell lines. Nature.

[33] Halama A, Horsch M, Kastenmüller G (2016). Metabolic switch during adipogenesis: from branched chain amino acid catabolism to lipid synthesis. Arch Biochem Biophys.

[34] Halama A, Riesen N, Möller G (2013). Identification of biomarkers for apoptosis in cancer cell lines using metabolomics: tools for individualized medicine. J Intern Med.

[35] Hansler A, Chen Q, Ma Y, Gross SS (2016). Untargeted metabolite profiling reveals that nitric oxide bioynthesis is an endogenous modulator of carotenoid biosynthesis in Deinococcus radiodurans and is required for extreme ionizing radiation resistance. Arch Biochem Biophys.

[36] Evans AM, DeHaven CD, Barrett T (2009). Integrated, nontargeted ultrahigh performance liquid chromatography/electrospray ionization tandem mass spectrometry platform for the identification and relative quantification of the small-molecule complement of biological systems. Anal Chem.

[37] DepMap Data Downloads. In: DepMap, Broad DepMap 21Q3 Public. figshare. Dataset. https://depmap.org/portal/download/all/?release=DepMap+Public+21Q3&file=Achilles_gene_effect_CERES.csv. Accessed 22 Nov 2021

[38] Van Der Meer D, Barthorpe S, Yang W (2019). Cell Model Passports-a hub for clinical, genetic and functional datasets of preclinical cancer models. Nucleic Acids Res.

[39] Xia J, Sinelnikov IV, Han B, Wishart DS (2015). MetaboAnalyst 3.0-making metabolomics more meaningful. Nucleic Acids Res.

[40] Saeed AI, Sharov V, White J (2003). TM4: a free, open-source system for microarray data management and analysis. Biotechniques.

[41] Egeblad M, Werb Z (2002). New functions for the matrix metalloproteinases in cancer progression. Nat Rev Cancer.

[42] Fang X, Cai Y, Liu J (2011). Twist2 contributes to breast cancer progression by promoting an epithelial-mesenchymal transition and cancer stem-like cell self-renewal. Oncogene.

[43] Powell E, Piwnica-Worms D, Piwnica-Worms H (2014). Contribution of p53 to metastasis. Cancer Discov.

[44] Pérez-Tenorio G, Stål O, Arnesson LG (2002). Activation of AKT/PKB in breast cancer predicts a worse outcome among endocrine treated patients. Br J Cancer.

[45] Whyte J, Bergin O, Bianchi A (2009). Key signalling nodes in mammary gland development and cancer. Mitogen-activated protein kinase signalling in experimental models of breast cancer progression and in mammary gland development. Breast Cancer Res.

[46] Sethi N, Dai X, Winter CG, Kang Y (2011). Tumor-derived JAGGED1 promotes osteolytic bone metastasis of breast cancer by engaging notch signaling in bone cells. Cancer Cell.

[47] Sanchez-Vega F, Mina M, Armenia J (2018). Oncogenic signaling pathways in the cancer genome atlas. Cell.

[48] Pavlova NN, Thompson CB (2016). The emerging hallmarks of cancer metabolism. Cell Metab.

[49] Gillies RJ, Robey I, Gatenby RA (2008). Causes and consequences of increased glucose metabolism of cancers. J Nucl Med.

[50] Wittig R, Coy JF (2007). The role of glucose metabolism and glucose-associated signalling in cancer. Perspect Medicin Chem.

[51] Zastre JA, Sweet RL, Hanberry BS, Ye S (2013). Linking vitamin B1 with cancer cell metabolism. Cancer Metab.

[52] Tarragó-Celada J, Cascante M (2021). Targeting the metabolic adaptation of metastatic cancer. Cancers (Basel)..

[53] Peng M, Yang D, Hou Y (2019). Intracellular citrate accumulation by oxidized ATM-mediated metabolism reprogramming via PFKP and CS enhances hypoxic breast cancer cell invasion and metastasis. Cell Death Dis.

[54] Payen VL, Porporato PE, Baselet B, Sonveaux P (2016). Metabolic changes associated with tumor metastasis, part 1: Tumor pH, glycolysis and the pentose phosphate pathway. Cell Mol Life Sci.

[55] Wei Y, Wang D, Jin F (2017). Pyruvate kinase type M2 promotes tumour cell exosome release via phosphorylating synaptosome-associated protein 23. Nat Commun.

[56] Tarragó-Celada J, Foguet C, Tarrado-Castellarnau M (2021). Cysteine and folate metabolism are targetable vulnerabilities of metastatic colorectal cancer. Cancers (Basel).

[57] Bonnomet A, Syne L, Brysse A (2012). A dynamic in vivo model of epithelial-to-mesenchymal transitions in circulating tumor cells and metastases of breast cancer. Oncogene.

[58] Chen LH, Liao CY, Lai LC (2019). Semaphorin 6A attenuates the migration capability of lung cancer cells via the NRF2/HMOX1 axis. Sci Rep.

[59] Liu J, Cao F, Li X (2021). ITIH5, a p53-responsive gene, inhibits the growth and metastasis of melanoma cells by downregulating the transcriptional activity of KLF4. Cell Death Dis.

[60] Thornalley PJ (1993). The glyoxalase system in health and disease. Mol Aspects Med.

[61] Tamori S, Nozaki Y, Motomura H (2018). Glyoxalase 1 gene is highly expressed in basal-like human breast cancers and contributes to survival of ALDH1-positive breast cancer stem cells. Oncotarget.

[62] Brand A, Singer K, Koehl GE (2016). LDHA-associated lactic acid production blunts tumor immunosurveillance by T and NK cells. Cell Metab.

[63] Wang D, Li W, Yin L (2020). Association of serum levels of deoxyribose 1-phosphate and S-lactoylglutathione with neoadjuvant chemotherapy sensitivity in patients with gastric cancer: a metabolomics study. Oncol Lett.

[64] Wu JY, Huang TW, Hsieh YT (2020). Cancer-derived succinate promotes macrophage polarization and cancer metastasis via succinate receptor. Mol Cell.

[65] Hamada-Kanazawa M, Narahara M, Takano M (2011). β-citryl-L-glutamate acts as an iron carrier to activate aconitase activity. Biol Pharm Bull.

[66] Schowen RL (1993) Principles of biochemistry, 2nd edn. In: Lehninger AL, Nelson DL, Cox MM. WH Free New York vol 70, p A223. Doi:10.1021/ed070pa223.1

[67] Röhrig F, Schulze A (2016). The multifaceted roles of fatty acid synthesis in cancer. Nat Rev Cancer.

[68] Ahmed N, Kidane B, Wang L (2021). Metabolic changes in early-stage non-small cell lung cancer patients after surgical resection. Cancers (Basel).

[69] Chen RR, Yung MMH, Xuan Y (2019). Targeting of lipid metabolism with a metabolic inhibitor cocktail eradicates peritoneal metastases in ovarian cancer cells. Commun Biol.

[70] Mossmann D, Park S, Hall MN (2018). mTOR signalling and cellular metabolism are mutual determinants in cancer. Nat Rev Cancer.

[71] Xiao F, Wang C, Yin H (2016). Leucine deprivation inhibits proliferation and induces apoptosis of human breast cancer cells via fatty acid synthase. Oncotarget.

[72] Tian Q, Yuan P, Quan C (2020). Phosphorylation of BCKDK of BCAA catabolism at Y246 by Src promotes metastasis of colorectal cancer. Oncogene.

[73] Ward MP, E. Kane L, A. Norris L, (2021). Platelets, immune cells and the coagulation cascade; friend or foe of the circulating tumour cell?. Mol Cancer.

[74] Kotkow KJ, Roth DA, Porter TJ (1993). Role of propeptide in vitamin k-dependent γ-carboxylation. Methods Enzymol.

[75] Schumacher D, Strilic B, Sivaraj KK (2013). Platelet-derived nucleotides promote tumor-cell transendothelial migration and metastasis via P2Y2 receptor. Cancer Cell.

[76] Thalor N, Singh K, Pujani M (2019). A correlation between platelet indices and preeclampsia. Hematol Transfus Cell Ther.

