# Supplementary figures and images for: Defining the landscape of metabolic dysregulations in cancer metastasis
Source: Clin Exp Metastasis. 2021 Dec 18;39(2):345–62. doi: 10.1007/s10585-021-10140-9 (PMC8971193; doi:10.1007/s10585-021-10140-9)

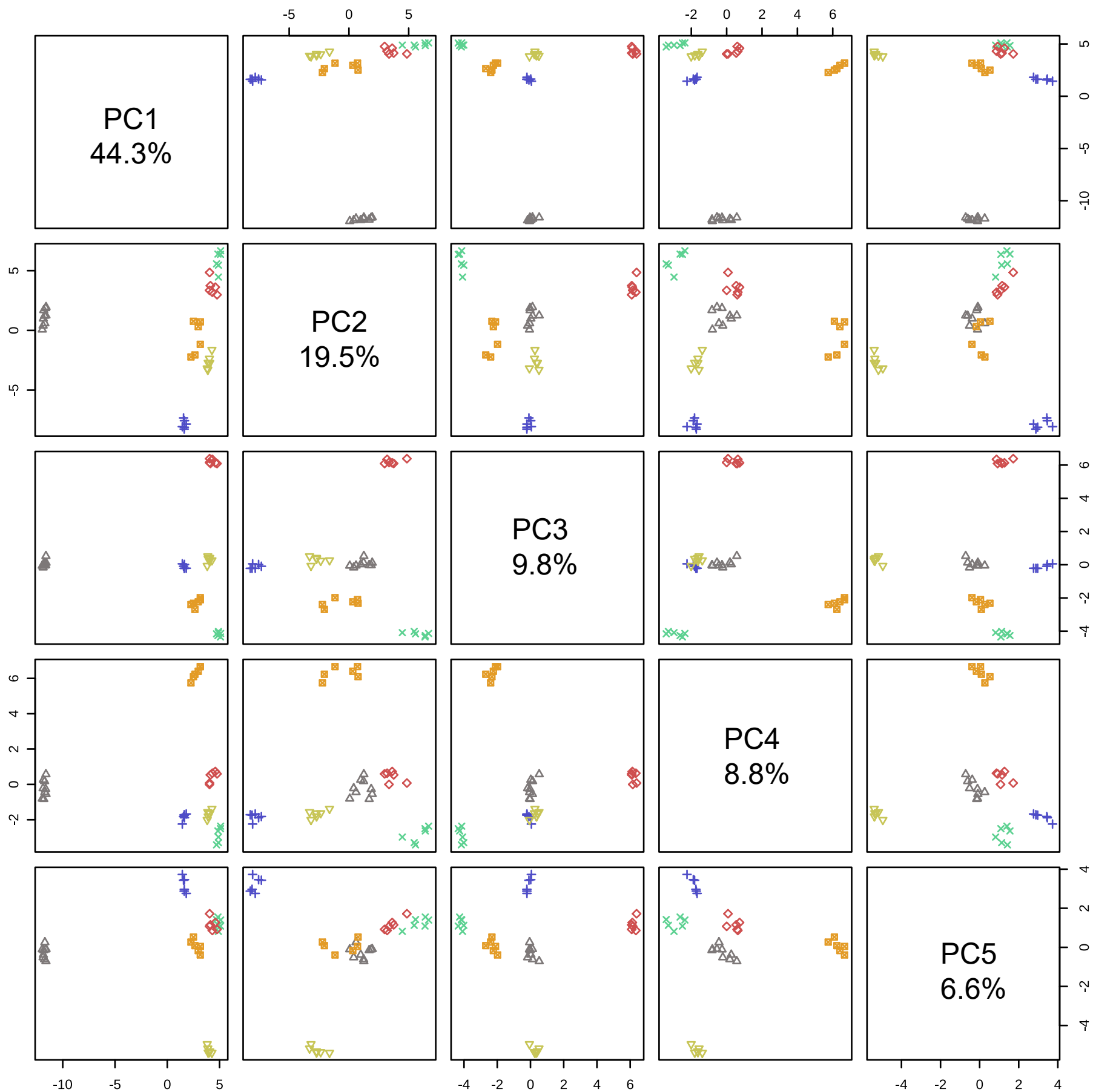

Supplement: Supplementary file 1 — Pairwise score plot for top five principal component (PC). Grey - hTERT-HME1; blue - BT549; green - HCC1143; read - MDA-MB-231; yellow - MDA-MB-436; orange - MDA-MB-468. Supplementary file1 (PDF 146 kb) [file 10585_2021_10140_MOESM1_ESM.pdf]

A)

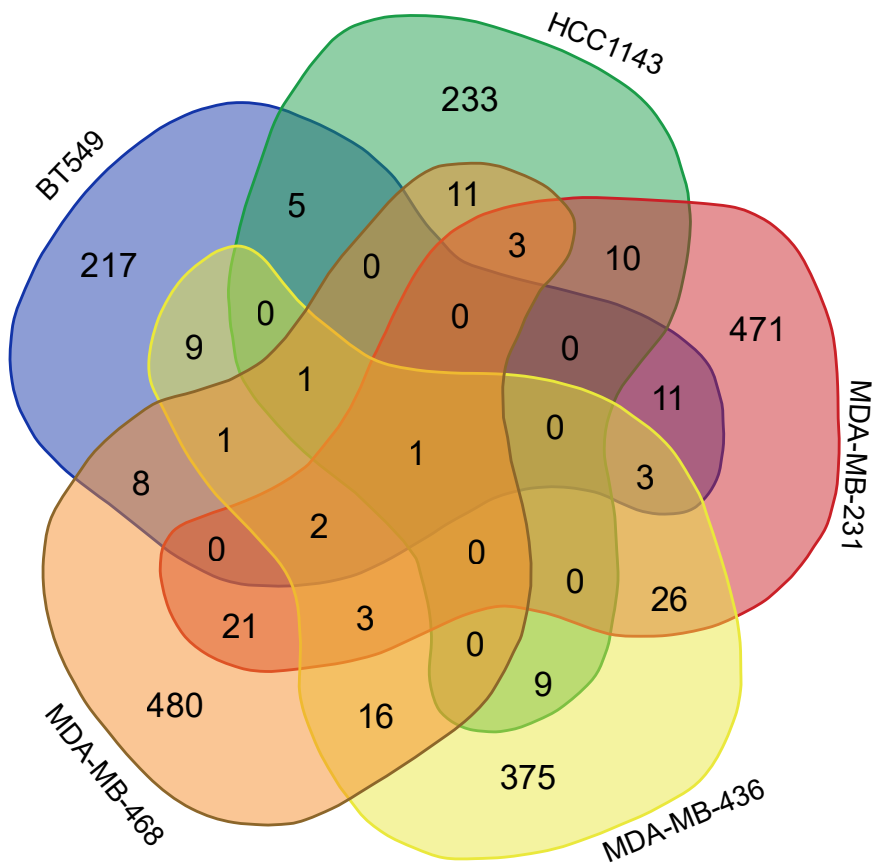

B)

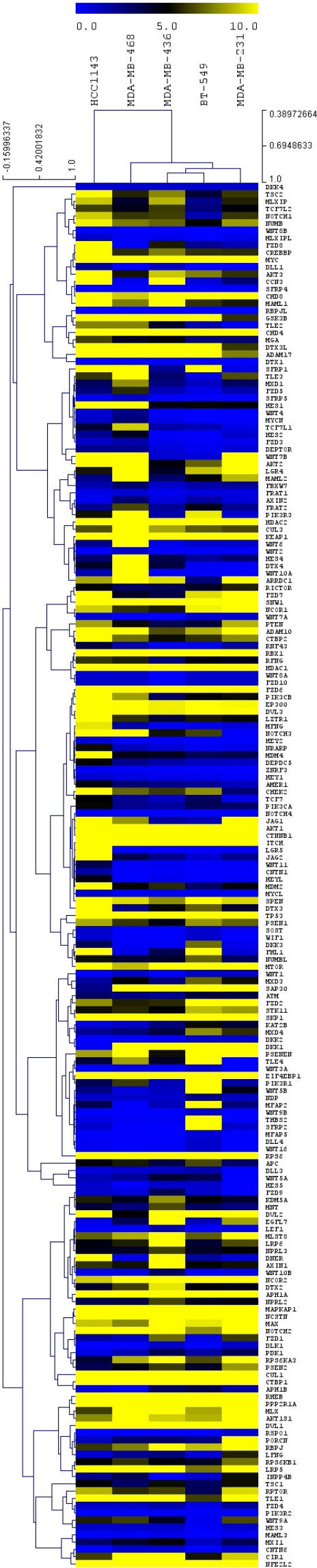

Supplement: Supplementary file 2 — A) Venn diagram showing overlap of mutated molecules across the five investigated TNBC cell lines. B) Hierarchical clustering (HCL) analysis of expression profiles of 190 genes across MYC, Notch, Nrf2, PI3K, Wnt and p53 pathways in five investigated cancer cell lines.. Supplementary file2 (PDF 587 kb) [file 10585_2021_10140_MOESM2_ESM.pdf]

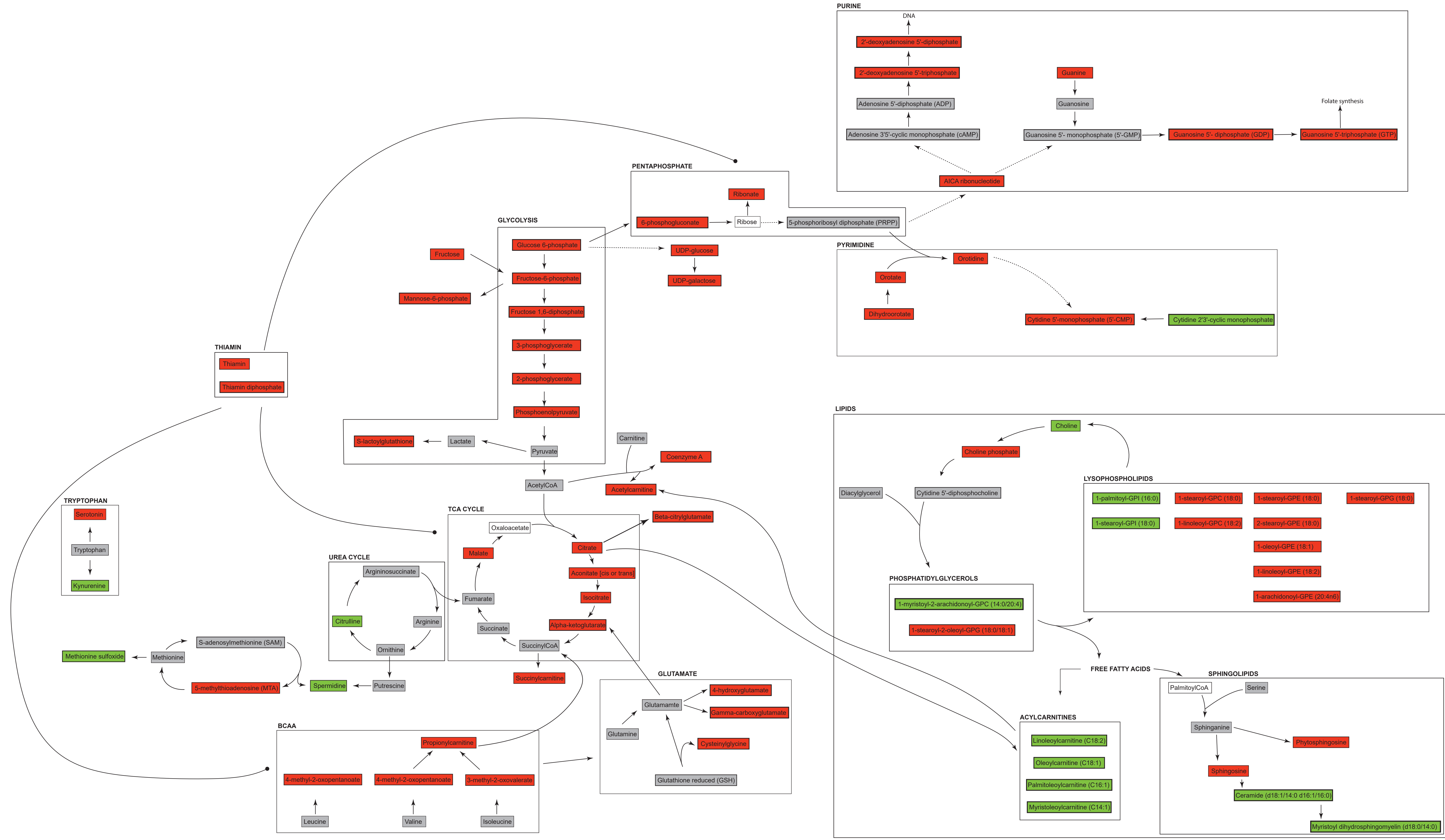

Supplement: Supplementary file 3 — Metabolic pathway depicting metabolic differences between HMP and LMP cell lines. Red – metabolites significantly elevated in HMP in comparison with LMP; Green – metabolites significantly decreased in HMP in comparison with LMP; Grey – measured metabolites not significantly different between HMP and LMP; White – metabolite not detected. Supplementary file3 (PDF 505 kb) [file 10585_2021_10140_MOESM3_ESM.pdf]

**A)**

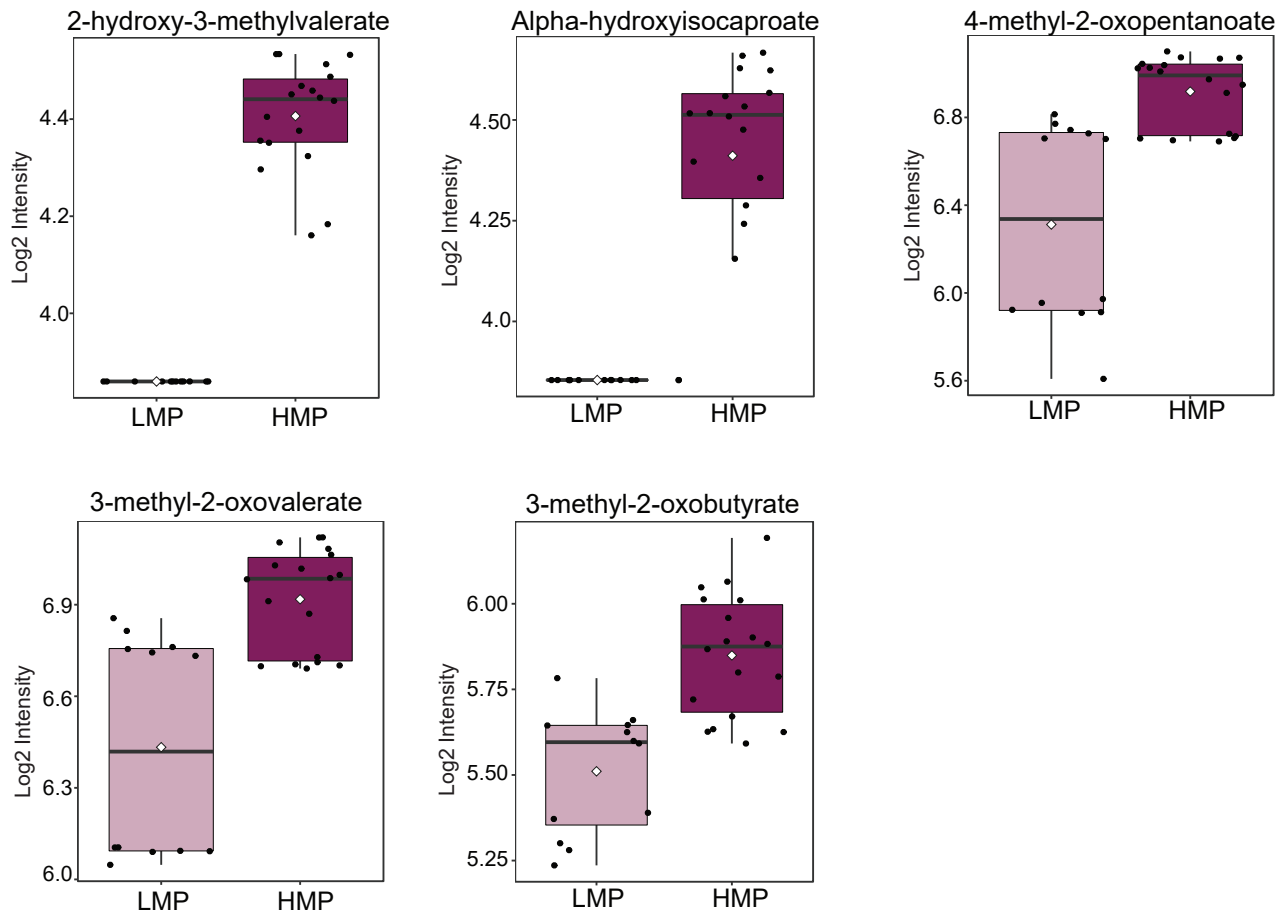

**B)**

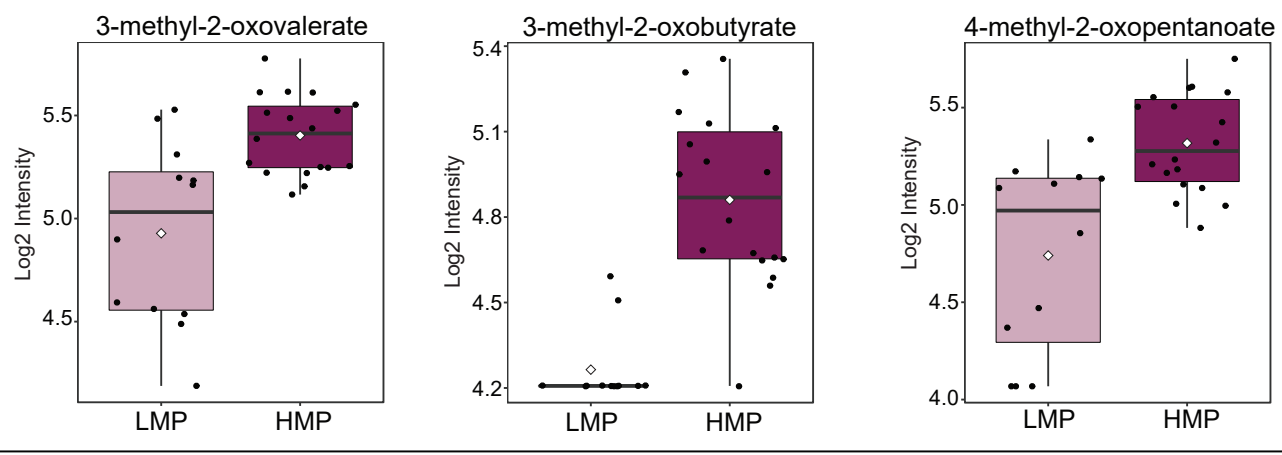

Supplement: Supplementary file 4 — . Box plots showing products of BCAA catabolism differentiating significantly HMP and LMP cell lines in A) growth medium and B) cells. Light and dark purple indicate cell lines with LMP and HMP respectively. Supplementary file4 (PDF 459 kb) [file 10585_2021_10140_MOESM4_ESM.pdf]

**A)**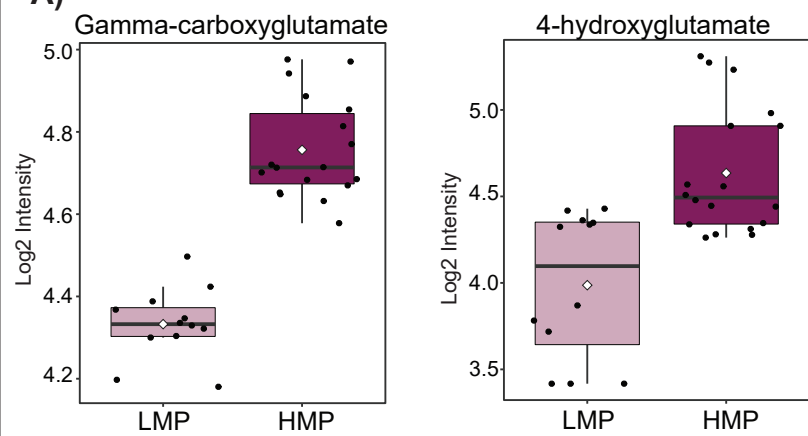**B)**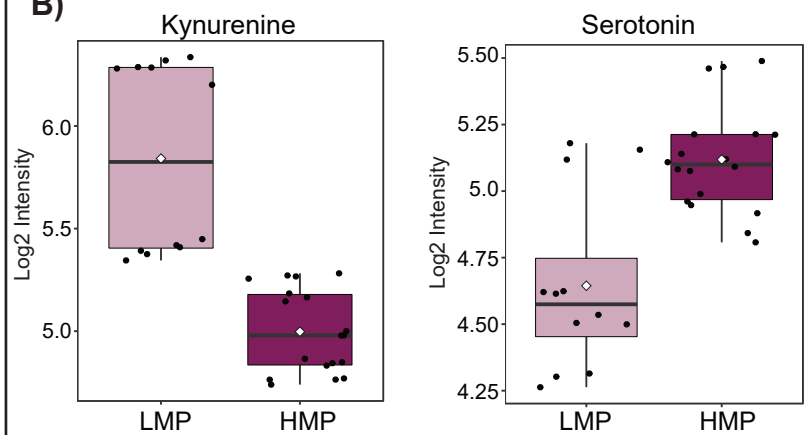**C)**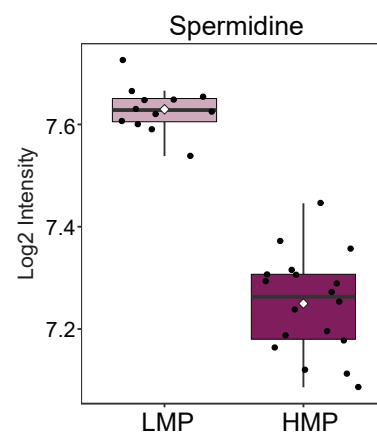

Supplement: Supplementary file 5 — Box plots showing metabolites differentiating significantly HMP and LMP cell lines involved in A) glutamate metabolism; B) tryptophane metabolism and C) polyamine metabolism. Light and dark purple indicate cell lines with LMP and HMP respectively. Supplementary file5 (PDF 437 kb) [file 10585_2021_10140_MOESM5_ESM.pdf]
